# SPECT/CT imaging features of cystic degeneration of the talus and their relation to pathological findings

**DOI:** 10.1186/s13018-022-03344-6

**Published:** 2022-10-12

**Authors:** Qitao Song, Xiantie Zeng, Haijing Huang, Lei Long, Jin Xu, Shuangshuang Cui, Zhonghai Li, Xinlong Ma

**Affiliations:** 1 Department of Nuclear Medicine, Tianjin Hospital, Tianjin, People’s Republic of China; 2 Department of Traumatology, Tianjin Hospital, Tianjin, People’s Republic of China; 3 Department of Pathology, Tianjin Hospital, Tianjin, People’s Republic of China; 4 Department of Orthopaedics, First Affiliated Hospital of Dalian Medical University, Dalian, People’s Republic of China

**Keywords:** Talus, Cystic degeneration, SPECT/CT, Diagnosis, Treatment

## Abstract

**Background:**

Osteochondral lesions of the talus (OLTs) are a common orthopedic condition. The image presentation is very similar to that of ischemic necrosis of the talus complicated by a talar neck fracture, but the two are very different lesions. When abnormalities in bone density (or signal) of the talar body (apex of the fornix) with concomitant bone defects and cystic changes are found on X-ray, computed tomography (CT), or magnetic resonance imaging, it is important to accurately determine the nature of the lesion and make a correct diagnosis for the treatment and prognosis of the patient. The purpose of this study was to explore the imaging features of three-phase single-photon emission computed tomography (SPECT)/CT images of cystic lesions of the talus.

**Methods:**

A total of 189 patients with chronic pain in the ankle joint suspected to be caused by cystic degeneration of the talus were enrolled. All patients underwent 99mTc-methyl diphosphonate (99mTc-MDP) three-phase SPECT/CT bone imaging and delayed scans in our hospital. The location, range of involvement, classification, CT value, and radioactivity uptake of the sclerotic areas of cystic lesions on the talus, and the continuity of the articular surface, were recorded. All recorded parameters were analyzed in comparison with pathological results.

**Results:**

Eighty-three percent (157/189) of the talar cysts were located on the medial fornix, largely involving the anterior middle part (43.27%), with larger cysts involving the posterior part (9.6%). Sixty-three percent (119/189) of the patients had type I lesions and 37% (70/189) had type II lesions. The articular surface of the medial dome of the talus was intact in all patients, but the subchondral bony articular surface was rough in 88% (166/189) of patients. The coincidence rate for the location, type, and range of involvement of cystic lesions with the pathological results was 87.83% (166/189). The mean CT value of the cystic lesions was 45 ± 15 HU (30–60 HU). The percentages of pathological chondrogenesis in high CT value ≥ 50 HU (19/70) and low CT value < 50 HU (51/70) groups were 89.47% (17/19) and 29.14% (15/51) (*χ*^2^ = 20.12, *p* < 0.001), respectively. The target/background ratio (T/B ratio) of the radioactivity-uptake area of the talus vault was 2.0 ± 0.5 (1.5–2.5). The percentages of pathological new trabecular bone in those with a T/B ratio ≥ 2.0 (157/189) and T/B ratio < 2.0 (32/189) were 82.80% (130/157) and 25.00% (8/32; *χ*^2^ = 45.08, *p* < 0.001), respectively.

**Conclusions:**

Three-phase bone imaging could identify damage of the talus caused by cystic degeneration, while delayed SPECT/CT images showed advantages for displaying bone microstructure, blood supplement, and bone metabolism when examining the location, range of involvement, classification, and repair of cystic lesions of the talus.

## Background

Osteochondral lesions of the talus (OLTs) are a common orthopedic condition. They have multiple causes and are characterized by cartilage damage, exfoliation, or subchondral osteonecrosis, and the formation of free osteochondral bodies. OLT was first described by Monro in 1856 as one of the causes of chronic pain in the ankle joint. In 1959, Berndt and Harty classified OLT into four types [1]: type 1 indicates simple compression injury, type 2 partial cartilage fracture, type 3 complete separation of cartilage fracture fragments without displacement, and type 4 displacement of fracture fragments. In 1993, Richard reported that imaging with SPECT and CT in the same patients revealed radiolucent defects and bone defects in the talus in 77% of patients. Berndt’s classification did not include this type of condition. Richard therefore added type 5 to Berndt’s classification, representing an area of intraosseous X-ray hypodense defect [2]. The image presentation of this type is very similar to that of ischemic necrosis of the talus complicated by a talar neck fracture, but the two are very different lesions. When abnormalities in bone density (or signal) of the talar body (apex of the fornix) with concomitant bone defects and cystic changes are found on X-ray, computed tomography (CT), or magnetic resonance imaging, it is important to accurately determine the nature of the lesion and make a correct diagnosis for the treatment and prognosis of the patient.

With continuous advances in medical imaging equipment, especially the implementation of single-photon emission computed tomography (SPECT)/CT, the rate of diagnosis of trauma and disease in the field of orthopedics has increased dramatically, and understanding of the nature of injury and disease has been elevated to a new level. In the case of osteochondral injuries of the talus, according to a multicenter study in Canada, one center diagnosed 13 patients in 5 years from 1981 to 1986, but the same group then diagnosed 79 patients over the next 5 years (1987–1992; a sixfold increase) and proposed new classifications [2].

To identify OLTs and ischemic necrosis in two different lesions, this study evaluated the blood supply and bone metabolism in osteochondral injury cystic lesions of the talus using SPECT/CT triphasic bone imaging and 16-row CT fusion images acquired on the same scanner. The included patients had chronic ankle pain without fracture-dislocation. A preliminary analysis of the characteristics and causes of osteochondral injury cystic lesions of the talus was made, making comparisons with the pathological findings of the surgical specimens.

## Methods

### Participants

A total of 189 patients with chronic pain in the ankle joint and with clinical suspicion of talar cystic degeneration were retrospectively enrolled from our hospital. The patients included 135 men and 54 women, with an age range of 23–66 years and a mean age of 41 years (Table 1). The inclusion criteria were as follows: (1) history of different degrees of ankle sprain; (2) clinical manifestation of chronic intermittent ankle pain combined with recurrent mild ankle swelling, weakness, and stiffness; (3) a VAS (visual analogue scale, a common and validated implementation to measure pain from 0–10, in which 0 means no pain and 10 means severe pain) score ≤ 3 and unaffected sleep at night; (4) uneven subchondral bone density in the medial (and in a few cases lateral) talar domes on plain X-ray radiographs and an unclear bony articular surface due to joint overlap and an unobvious narrowed joint space; and (5) imaging with a SPECT/CT three-phase bone scan. The exclusion criteria included: (i) patients with talar fractures; and (ii) patients with a history of long-term heavy alcohol consumption or hormone therapy. All patients underwent surgical circumferential drilling of the damaged cystic area of the talus in our hospital, and the cystic area was filled with autologous osteochondral column mosaic grafts. Intraoperative specimens were sent to pathology for definitive diagnosis. All participants gave informed consent for the use of their information for research purposes.

**Table 1:** Summary of demographic data in 189 patients

Variable	*n* (%)
Gender (male:female)	2.5:1
Age	
20–30	15 (8%)
31–40	67 (35%)
41–50	62 (34%)
51–60	31 (16%)
> 60	14 (7%)
Medical history	
Ankle internal rotation	161 (85%)
Ankle movement restricted	28 (15%)
Clinical symptoms	
Mild swelling of the ankle	42 (22%)
Medial ankle pain	168 (89%)

### ^***99m***^***Tc-MDP SPECT/CT scans***

A Discovery NM670 SPECT/CT scanner (GE Healthcare, USA) equipped with a low-energy high-resolution parallel-hole collimator was used. The patients were injected with 740 MBq (volume < 1 ml) of 99mTc-methyl diphosphonate (99mTc-MDP) via the elbow vein. The 99mTc-MDP was produced by HTA Co., and had a radiochemical purity of more than 95%.

**Fig. 1:**
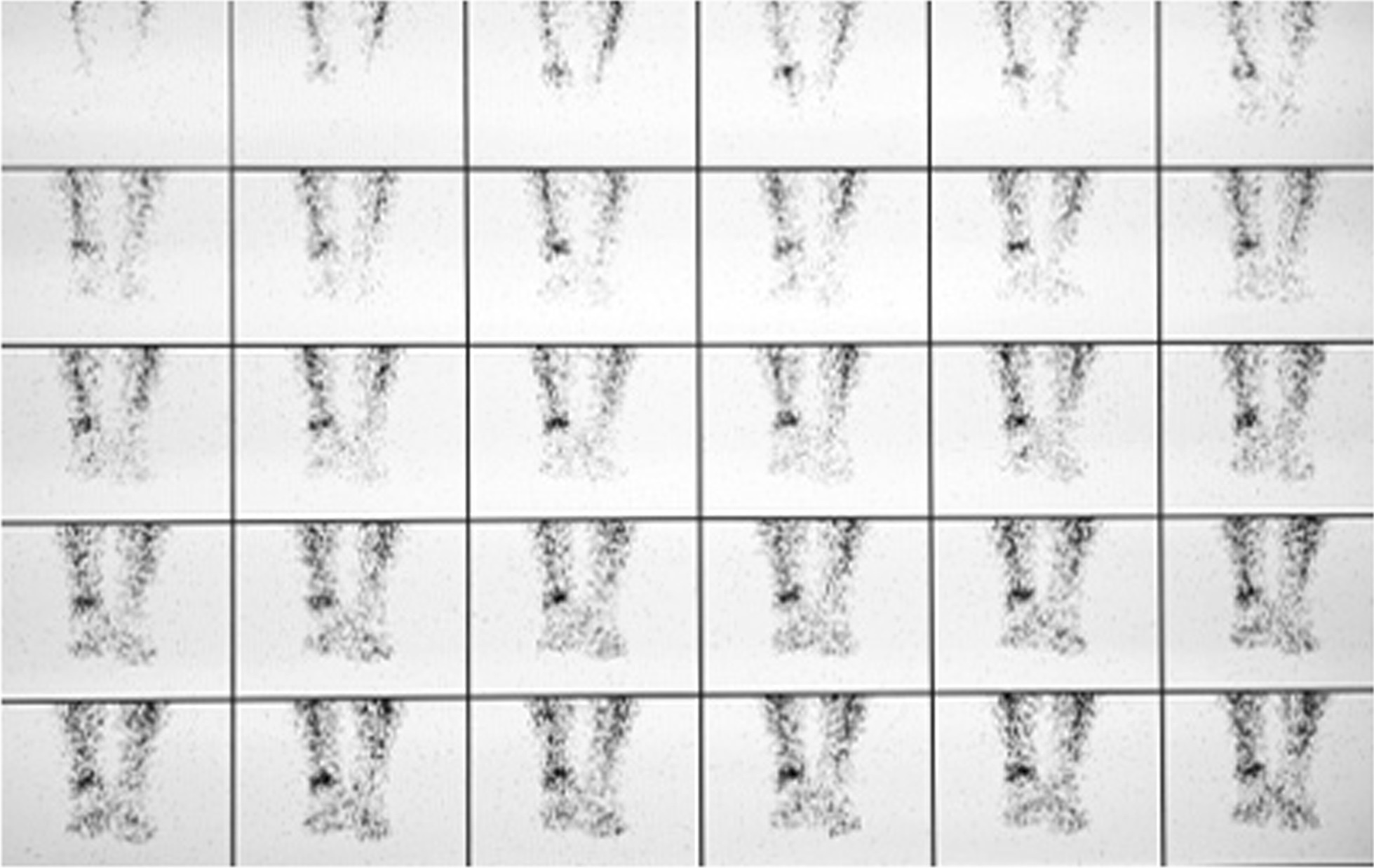
Blood flow in the large artery of the lower leg. The tracer concentration on the affected side is higher than that on the healthy side, suggesting that the lower leg on the affected side is congested

**Fig. 2:**
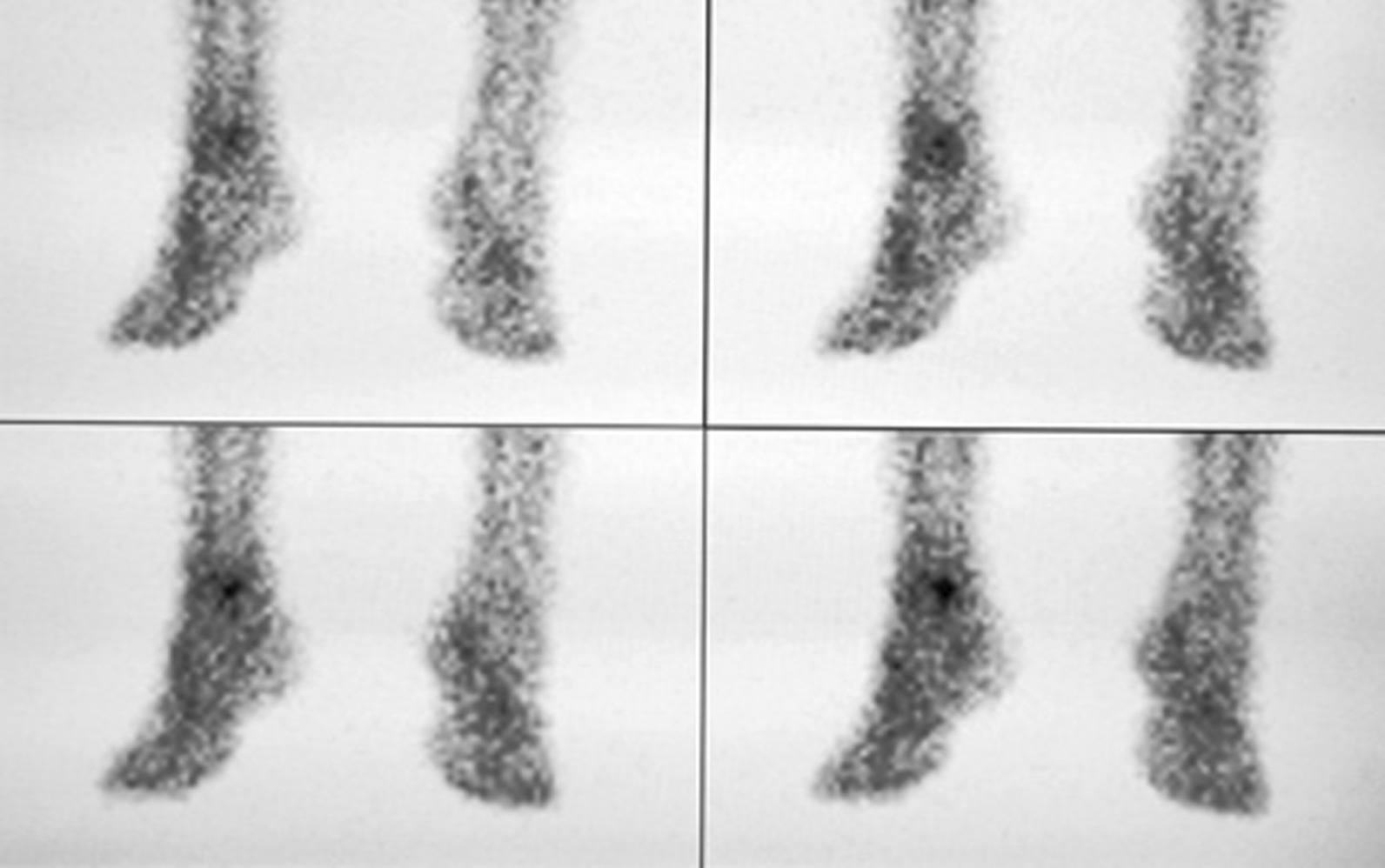
Blood pool phase. A dotted tracer concentration is visible in the affected ankle, and the overall tracer concentration on the affected side is enhanced compared with the healthy side, showing a bruised state

**Fig. 3:**
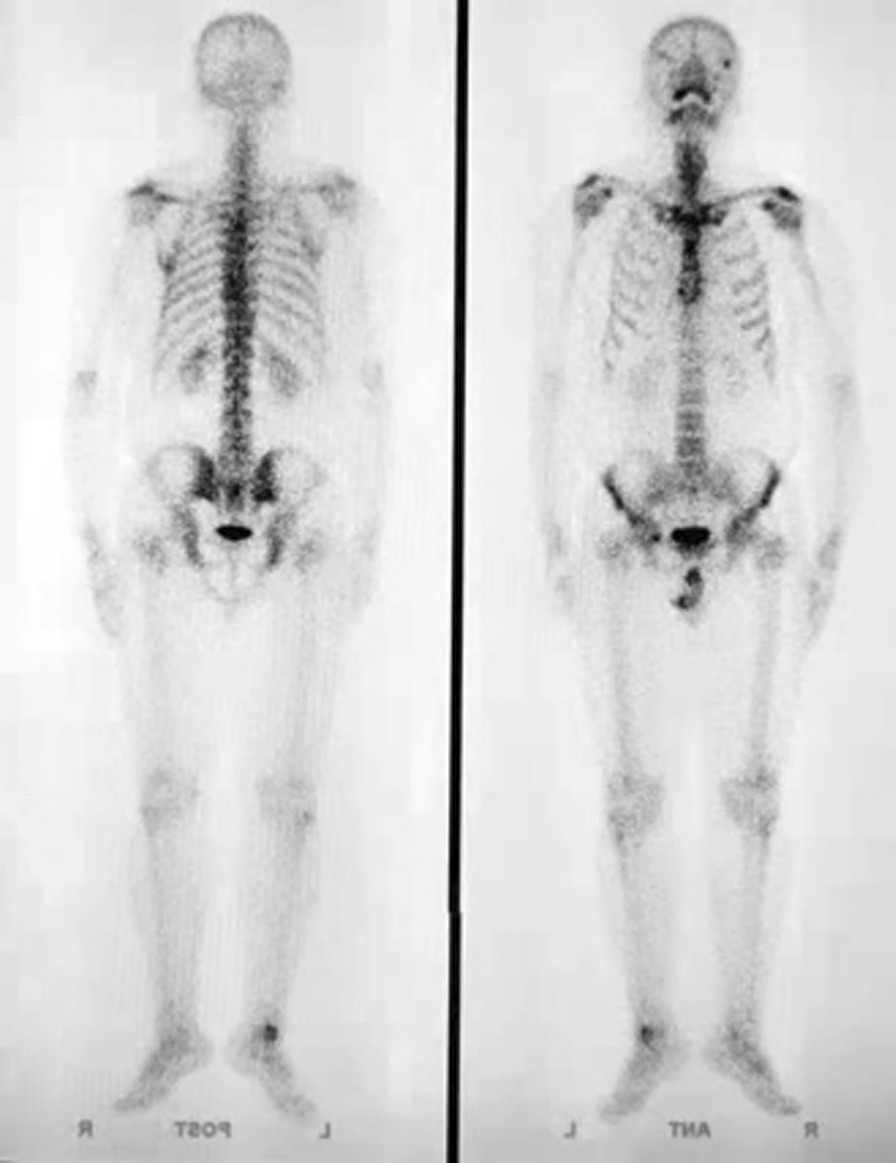
Delayed phase (static phase). A punctate tracer concentration is visible in the affected ankle joint, which suggests active local bone metabolism

Anterior and posterior planar images of the perfusion phase, blood pool phase, and delayed phase, and CT tomography images of the delayed phase of the ankle, were acquired bilaterally. The SPECT acquisition parameters were as follows: energy peak 140 keV, window width 20%, matrix 128 × 128, Zoom 1.25. The perfusion phase (Fig. 1) acquired immediately after intravenous injection of the imaging agent used a continuous acquisition at 2 s/frame for 60 s. The arterial blood flow was observed to determine whether local ‘congestion’ or ‘ischemia’ was present. The blood pool phase (Fig. 2) was acquired after the end of the perfusion phase using 1 min/frame for 10 min of continuous acquisition. The soft tissue venous blood flow was observed to determine whether it showed local ‘bruising’. The delayed phase (Fig. 3) was acquired 2 h after the injection of the imaging agent, with the acquisition count reaching 1 × 10^5^ kcts (kilocounts). In this phase, the tracer in the soft tissue faded while the tracer in the bone showed clearly, thereby reflecting the metabolic condition of the bone. The CT acquisition parameters were as follows: 120 kV tube voltage, 150 mA, layer thickness 10 mm, and pitch 1.0.

### Image analysis

To further clarify the extent of ischemia and necrosis in the damaged talus, we used 99mTc-MDP SPECT/CT triple-phase bone scanning and simultaneous fusion imaging. The first imaging method was to clarify whether the inhomogeneous bone density area shown on X-ray coincided with an area of abnormal tracer uptake. The second method was to allow visualization of whether there was an absence of tracer distribution or an abnormal concentration in the cystic area of the talus. The SPECT/CT image is on a black to white spectrum. The degree of color is determined by the concentration level of radioactive nuclide. On the white end of the spectrum, no radioactive nuclide exists, and on the black end, the radioactive nuclide concentrates a lot. If the local tomographic image was white, the area could be considered ischemic. Otherwise, if the local tomographic image was black, it reflected abundant blood flow and high bone metabolism in the lesion area.

**Fig. 4:**
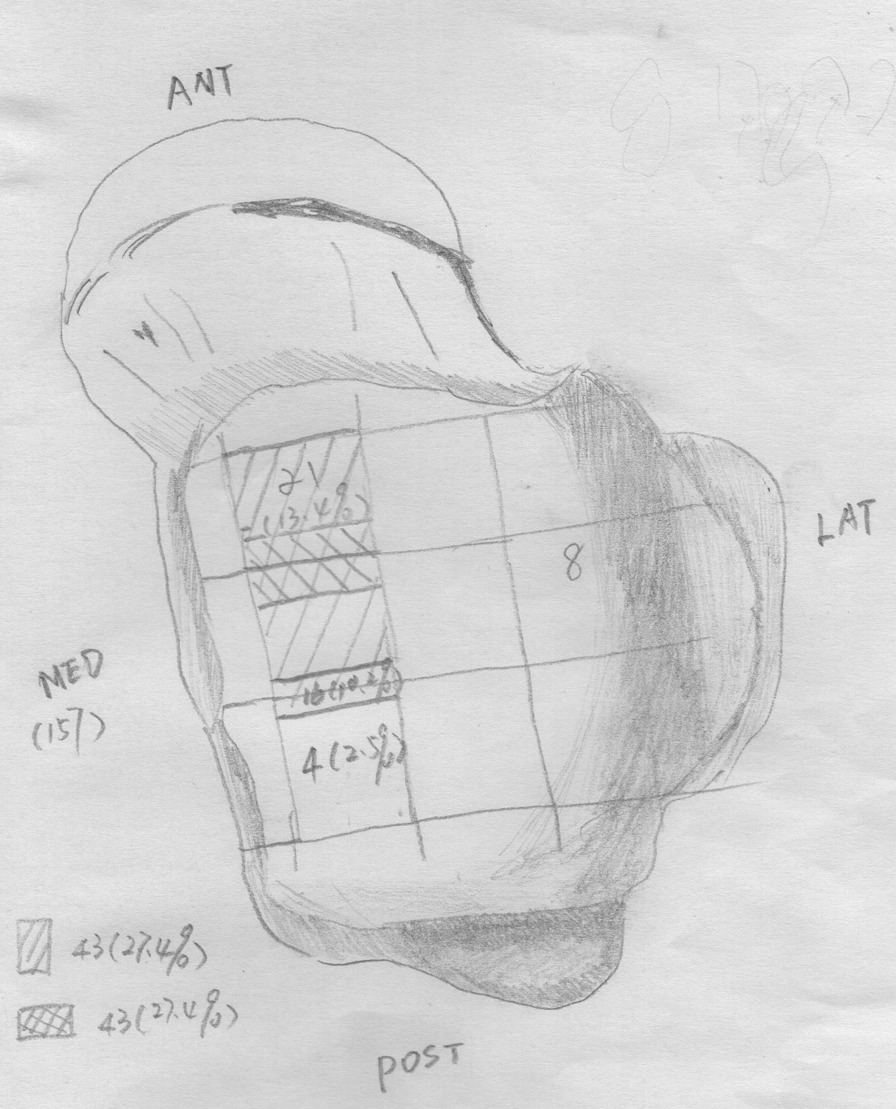
Hand drawing of the Elias subdivision of the talar dome. The lesions in this group are mainly concentrated in the medial mid-anterior part of the talar dome

**Fig. 5:**
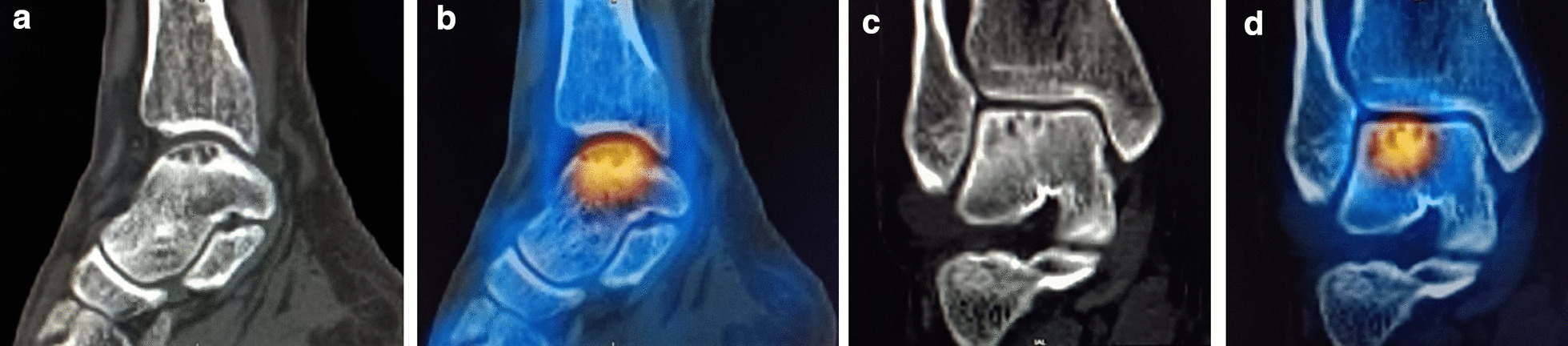
A 57-year-old man with left ankle pain for more than 5 years, history of a minor sprain, and other medical histories. CT sagittal (**a**) and coronal (**c**) images show multiple consecutive small bursae with sclerosis of the surrounding bone visible in the subchondral bone of the talar fornix. This is consistent with a type I cystic lesion. SPECT/CT fusion images (**b,d**) show that the area of tracer concentration in the talar fornix corresponds to the cystic area and the surrounding sclerotic bone

**Fig. 6:**
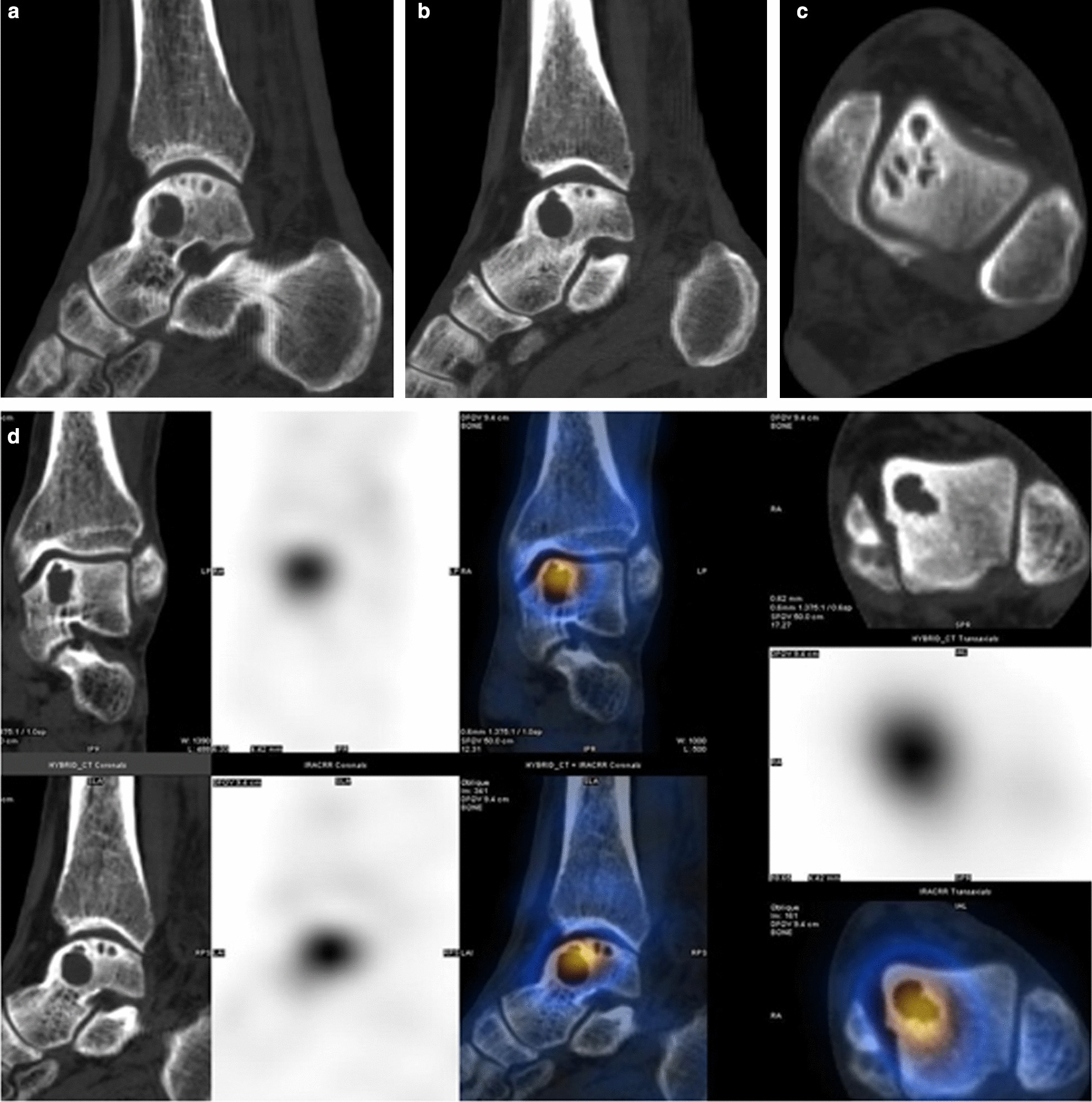
A 65-year-old man who had left ankle pain for 4.5 years that was then aggravated for 1 week. The fusion image shows that the lateral fornix of the left talus was locally fractured and the articular surface was not smooth. Multiple small capsular hypodense shadows can be seen underneath it, with obvious sclerosis of the edges. A larger cystic hypointense shadow can be seen below the anterior part of the small cyst, and a narrow channel can be seen connecting with the articular surface. The local bone cortex was not continuous. The area of anomalous tracer concentration in the left talus corresponds to the area of foraminal cystic degeneration (Type II cystic lesion). **a** Sagittal type II large cyst formation; **b** large cyst communicating with the articular surface; **c** multiple small cysts in an axial position; **d** SPECT/CT fusion image

The SPECT/CT images were fused and analyzed using a Xeleris workstation (GE Healthcare) according to the following steps. (1) The location of the talar fornix lesion was observed and recorded according to the Elias nine-pattern scheme (Fig. 4) [3]. (2) The extent of involvement of the talar cystic lesion was clarified. (3) The lesion was classified according to the extent of the cystic parts and the number of lesions into type I or II. Type I consisted of several independent and staggered bursae under the articular surface of the fornix, which were separated and surrounded by a bony sclerotic zone (Fig. 5). Type II was developed from type I, and represented a large thin-walled spherical cystic lesion. Cystic lesions are approximately 10–15 mm in diameter, with thin-walled sclerosis visible at the edges and a large cyst located deeply, often below a small cyst and anterior to the middle of the medial fornix, connecting to the small cyst or extending upward to the articular surface through a narrow channel (Fig. 6). Type II is more symptomatic and has a poorer prognosis. (4) The cystic lesion was measured on CT to indirectly reflect the tissue composition within the lesion area [4–6]. (5) The bone continuity of the articular surface above the cystic area was observed in all directions on sagittal, coronal, and transverse views. (6) The hematologic metabolism of the sclerotic zone was observed, and semi-quantitative analysis of the non-uniformly-thickened sclerotic zone formed by the deposition of new bone minerals around the subchondral bone cystic lesion was used to measure the ratio of the tracer concentration of the affected side to that of the corresponding healthy side (T/B ratio). When determining the target to background uptake, average count is measured: The affected side is T, and the corresponding healthy side is B.

### Pathological analysis

**Fig. 7:**
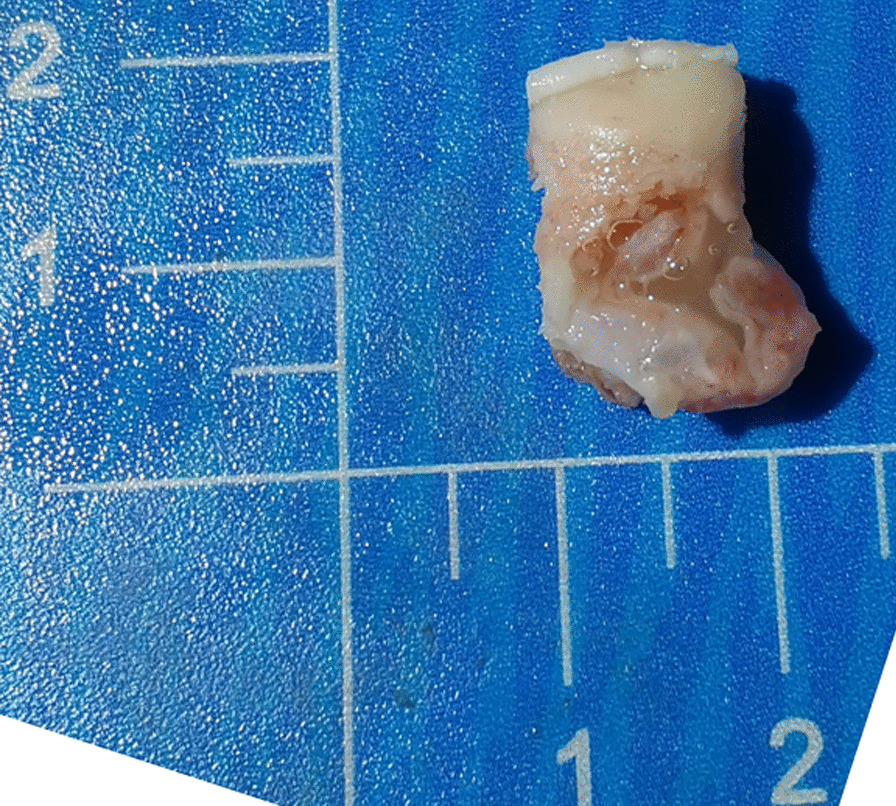
Gross specimen with subchondral bone defect and a mucus-like material residue visible at the cystic lesion

**Fig. 8:**
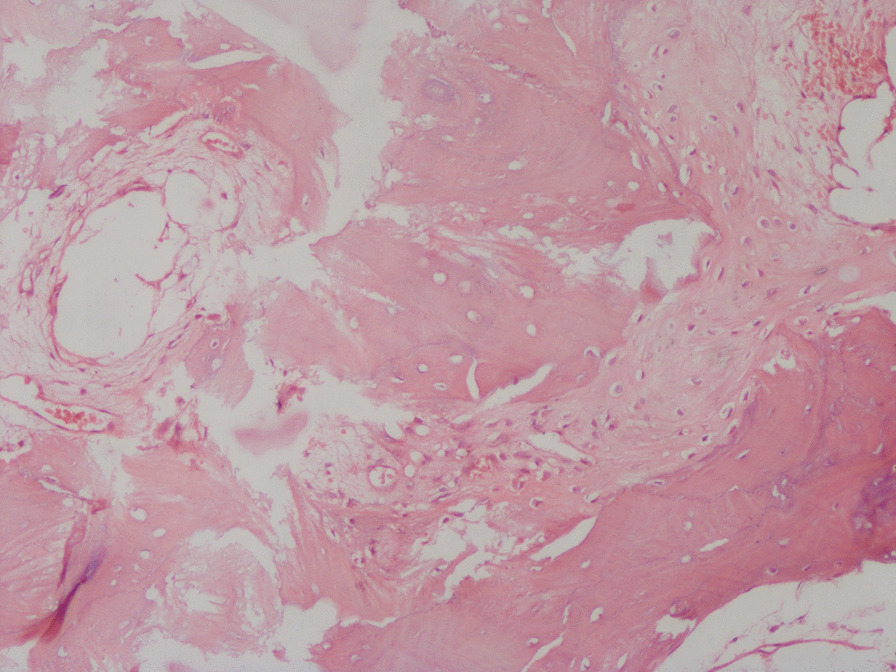
Hematoxylin and eosin-stained slide at ×100 magnification. Part of the bone traps was empty, osteocytes had disappeared, fibroblasts proliferated, collagen fibers were abundant, inflammatory cells were gathered, and fibroblastic granulation tissue grew in between the necrotic bone trabeculae

**Fig. 9:**
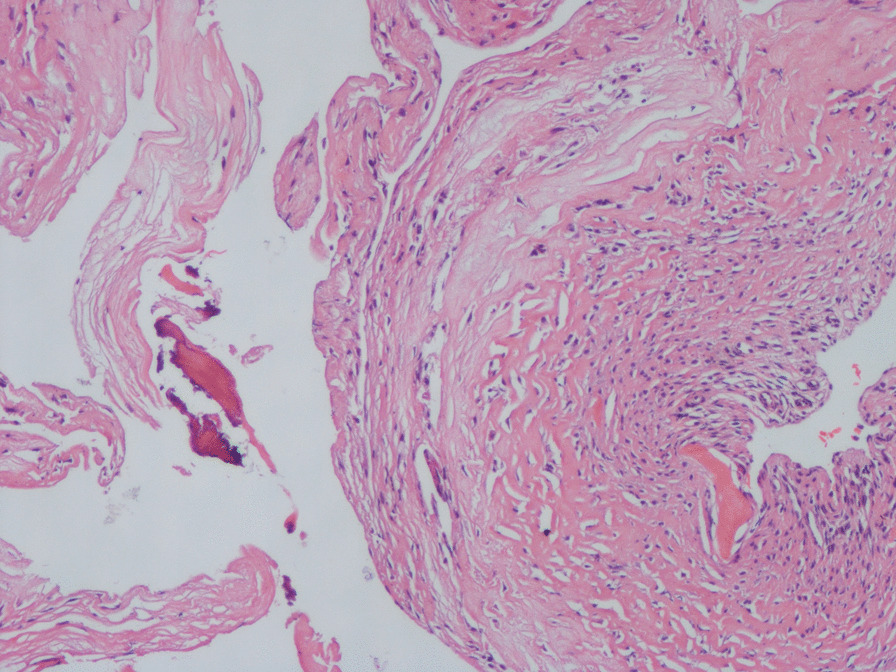
Scattered bone-like stroma is visible between the hyperplastic granulation tissue of the tortuous fibrous cyst wall on a hematoxylin and eosin-stained slide at ×100 magnification

**Fig. 10:**
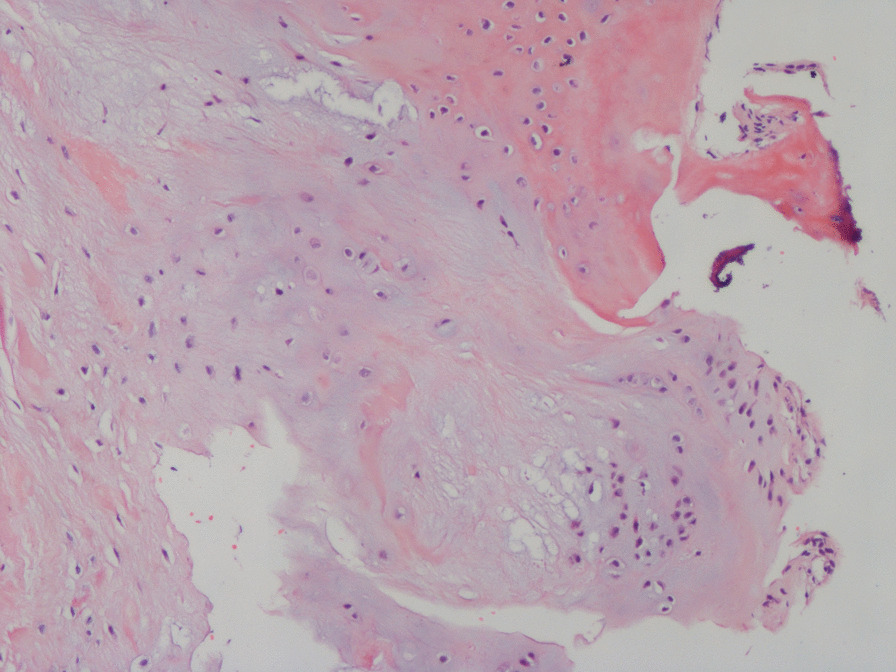
Hematoxylin and eosin-stained slide at ×100 magnification. Chondrocytes, and chondrogenesis with ossification are visible in the thickened cyst wall

Specimens were taken intraoperatively and processed to observe the morphology and visible tissue types. Then, microscopic analyses were performed. The gross specimens showed focal wear thinning of the cartilage in the talar fornix with visible fractures and partial subchondral bone defects. Mucus-like material was found in the larger cystic lesions (Fig. 7). Microscopically, some of the bone traps were empty, osteoblasts were missing, fibroblasts were proliferating, collagen fibers were abundant, inflammatory cells were aggregating, and fibroblastic tissue was visible growing in between the necrotic bone trabeculae (Fig. 8). Folded and tortuous fibrous cyst walls were seen in intact specimens, and scattered bone-like stroma was visible between the hyperplastic granulation tissue of the cyst walls (Fig. 9). Chondrocytes and chondrogenesis with ossification were seen in the partially thickened cyst wall (Fig. 10).

### Statistical analysis

SPSS 22.0 was used for statistical analysis. Measurement data are expressed as mean ± standard deviation (SD). Comparisons between different SPECT/CT signs and pathological diagnostic results were analyzed using Chi-square tests. A value of *p* < 0.05 was considered statistically significant.

## Results

### SPECT/CT imaging results

#### Features of three-phase bone plane imaging

As shown in Figs. 1, 2 and 3, the signal from the soft tissue of the affected ankle joint was higher than that of the healthy side in the perfusion phase, blood pool phase, and delayed phase in 100% (189/189) of patients, and the talus showed abnormal concentration of radioactivity.

### Characteristics of the delayed SPECT/CT fusion image

*Site of the talar cystic lesions* Eighty-three percent (157/189) of the cystic lesions were located in the medial fornix, 4% (8/189) in the lateral fornix, and 13% (24/189) were symmetrically distributed in the medial fornix of both ankles.

*The extent of cystic lesions in the talus* The cystic lesions were mostly located in the medial fornix of the talus, with the middle anterior 1/3 of the medial fornix being the most commonly involved site, with 27.4% (43/157) of cases being anterior to zone 4 of the ninth compartment of the Elias pattern (Fig. 4). The middle and anterior part of the medial fornix were both involved in 27.4% (43/157) of patients. In 5.7% (9/157) of the patients, larger lesions extended from the anterior to the posterior part of the medial fornix of the talus. The anterior, middle, and posterior parts were involved separately in 13.4% (21/157), 13.4% (21/157), and 12.7% (20/157) of the patients, respectively.

*Subarticular facet cystic lesion types* One hundred and nineteen cases were of type I (119/189, 63%) and 70 cases were of type II (70/189, 37%). The mean CT value of the cystic lesion area was 110 ± 30 HU (80–140 HU), with the mean value of large cysts being 45 ± 15 HU (30–60 HU) and that of small ones 180 ± 40 HU (130–230 HU).

S*ubchondral bone articular surface continuity* Eighty-eight percent (166/189) of cases showed a thicker sclerotic layer of type I cystic lesions, rough subchondral bone articular surface, and faintly visible chap-like small bone fractures, while almost all (100%) type II large cystic lesions were directly connected to the articular surface or were connected by narrow channels. The fractures were more clearly visible in type II lesions than in type I lesions, and in the larger cases, the apical cortical fissures were wider or formed bone defects. Regardless of the size of the cystic lesions, no collapse of the medial talar fornix articular surface was observed. The curvilinear pattern of the talar fornix articular surface remained. The bony articular surface could be worn and thinned.

*Hemometabolism of the sclerotic zone* All of the patients (189/189) showed a distinctly abnormal radioactivity concentration in the sclerotic zone around the cystic lesion, which showed a localized high level of hematologic and bone metabolism. The most obvious concentration was located in the anterior part of the talar fornix (the stress point of the dorsal extension of the tibial talar joint) in 81% (153/189) of cases.

*Semi-quantitative analysis* The mean T/B ratio of the radioactivity concentration zone of the affected talar fornix was 2.0 ± 0.5 (range: 1.5–2.5).

### Correlation of SPECT/CT features and pathological results

The SPECT/CT imaging of talar cystic lesions showed 88% (166/189 cases) agreement with the intraoperative visual observation and pathologic gross morphology in terms of cystic fractionation, cyst location, and extent of injury involvement. In the remaining 23 cases that did not show a match, the talar dome was not completely exposed intraoperatively or subchondral bone fractures were too small to be visible on CT images.

When measuring type II lesions, twenty-seven percent (19/70) of the cystic lesions showed high CT values (≥ 50 HU), and 89% of these cases (17/19) showed mucinous fibroblastic granulation with chondrogenesis and a small amount of osteoid stroma. In two cases there was a residual bone crest in the cystic lesion. In the low-CT group, 30% of cases (15) had a small amount of mucinous fibroblastic granulation with chondrogenesis, whereas the remaining 36 cases had only synovial tissue and a small amount of mucus. A significant difference in chondrogenesis was found between these two groups (*χ*^2^ = 20.123, *p* < 0.001; Table 2).

**Table 2:** Correlation analysis of CT values of the talar cystic area with pathological chondrogenesis

CT value	Chondrogenesis ( −)	Chondrogenesis ( +)	*χ*^2^	*p*
< 50Hu	36	15	20.123	0.000
≥ 50Hu	2	17		

The T/B ratio in the high-uptake area of the talar fornix was larger than 2.0 in 83% (157/189) of cases, with abnormally high values at the sclerotic zone around the cystic lesion. One hundred and thirty cases showed degeneration of the articular hyaline cartilage matrix with the formation of new bone trabeculae at the edge of the cystic wall. Seventeen cases (32/189) had a T/B ratio < 2.0 in the high uptake area of the talar fornix, with mild sclerosis of the thin cystic wall. The pathological study showed that only eight cases developed new bone trabeculae, and the difference between the two groups was significant (*χ*^2^ = 45.08, *p* < 0.001; Table 3).

**Table 3:** Correlation analysis of T/B ratio with pathological new bone trabeculae at high uptake area

New bone trabeculae	T/B < 2.0	T/B ≥ 2.0	*χ*^2^	*p*
Negative	24	27	45.077	0.000
Positive	8	130		

T/B is the ratio of the radioactivity of the affected side to the healthy side in the high uptake area of the talar dome

## Discussion

The recognition and treatment of osteochondral injuries of the talus have been the subjects of worldwide debate [7–9]. The exact history of the injury is hardly known in most cases, and medical records rarely document it because early asymptomatic injury can be easily overlooked [10]. Studies have reported that 25–50% of patients with ankle sprains will experience chronic long-term ankle pain for several years after the initial injury (sprain) [11–13]. Therefore, the exact incidence of osteochondral injuries remains unknown, with only one study stating that 27 patients suffered from osteochondral injuries per 100 000 people between 1998 and 2008 [14]. Draper et al. found that the incidence of chronic injuries to the talus due to ankle instability was up to 50% [15, 16].

Patients with chronic pain in the ankle joint are common in clinical practice [17]. When the density of the talus is altered on the first radiograph, the attending physician often habitually diagnoses osteonecrosis of the talus. In the medical literature, the terms osteonecrosis and ischemic necrosis have been used interchangeably for bone death due to impaired circulation [18]. This necrosis is often considered to be caused by a circulatory disorder. Thus, osteonecrosis of the talus seems to be a shorthand or synonym for ischemic necrosis of the talus. In contrast, the main clinical causes of ischemic necrosis of the talus are interruption of arterial blood supply and avulsion of the periosteal vascular network of the talar articular surface due to fracture of the talar neck and ankle dislocation. In our cases, there was no history of fracture or dislocation, and the local blood supply, bone metabolism, and pathology of the lesion showed different features from those of ischemic necrosis of the talus.

Three-phase bone imaging showed no ischemia in and around the cystic area of the talus. Compared with the healthy side, the cystic area showed significant concentrations of tracer and abundant blood flow. The blood flow and blood pool phases of the three-phase bone imaging represent the state of blood supply to the lower extremity (especially around the ankle joint) in the arterial and venous phases, respectively. The arterial phase of the affected side is congested, and the dorsalis pedis is patent without stenosis or occlusion, and slightly more congested than on the healthy side. The blood pool image is of the venous phase, with apparent concentrations of tracer at the talus, indicating stasis of blood flow in the venous phase. The static phase (the delayed phase) shows increased subarticular uptake, implying an active bone metabolism that extends over the entire medial or lateral aspect of the talus.

Different trauma mechanisms correspond to different injury sites. Some scholars believe that osteochondral injuries of the talus have a typical site of onset, namely the medial fornix or the lateral fornix of the talus, with the medial fornix being particularly common [19–21]. A study at Tong Ren Hospital in China found that medial injury accounted for 76% of injuries [22]. Elias et al. found that injuries located in the medial third were larger and deeper than those located elsewhere [3]. Cao et al. noted that deeper-position cystic changes in the medial subchondral talus were typical of patients over 60 years of age with talar cartilage injuries [23].

In this study, most of the patients had medial fornix injuries due to dorsal extension and internal rotation. In the more severe cases, the posterior part of the medial fornix was also involved, with almost no cases showing posterior involvement alone. In larger cases, the extent of injuries can extend from the anterior to the posterior part of the medial fornix of the talus. We conclude that cystic lesions typically begin from the anterior to the middle of the medial fornix, with the middle and anterior parts being the main areas of involvement. As the disease progresses, it continues to expand anteriorly and posteriorly. Distinguishing them from post-fracture talar necrosis, our cases, regardless of size, did not reveal nodal fracture or collapse of the talar fornix. The curvilinear morphology of the articular surface of the talar fornix remained, and the bony articular surface could be phenotypic but was never sunken. In contrast, ischemic necrosis of the talar articular surface often shows collapses of the necrotic bone tissue, loss of the normal morphology of the bony articular surface, and compression and flattening of the fornix.

The application of SPECT/CT, a new multimodality imaging method for patients with cystic lesions of the talus, allows assessment of the hemodynamic metabolic status of OLTs in real-time. The subchondral bone cystic lesion and surrounding area show the highest activity, whereas the rest of the talus presents a normal physiological bone metabolic zone [24–26]. The sclerotic zone is located at the junction of the lesion area and normal tissue, which is a zone rich in blood flow and new capillaries and shows active bone metabolism. An obvious concentration of tracer is located in the anterior part of the talar fornix and the stress point of the dorsal extension of the tibial talar joint, where the sclerotic zone is most concentrated, bone metabolism is most active, and blood flow is most abundant.

Simultaneous CT imaging showed alterations in the bone density of the talus, with the CT values varying greatly with the size of the cyst. This is because the smaller the cyst, the more it is affected by partial volume effects, with the measurement being subject to errors. Around subchondral bone cystic lesions, calcium salt deposits form unevenly-thickened sclerotic bands that provide strong mechanical support, especially in the weight-bearing zone of the tibial talar joint and at mechanical stress points. The sclerotic zone represents compensatory repair and an active response to injury. The joint is attempting to protect itself and restore the stability of its mechanical properties while restoring the original bone structure.

The functional imaging of blood metabolism on SPECT, the imaging of sclerotic zone morphology of CT, and the expression of multiple cellular components on pathology, comprehensively explain why collapse occurs in femoral head necrosis, but not in the articular surface of the talar vault. Although it is a weight-bearing joint like the hip and knee, the talus has a smaller weight-bearing area than the femur and carries almost the entire body’s mass. It is subject to more stress than the knee and hip and is more likely to be injured. Our SPECT/CT study confirmed that the blood supply to the affected limb was increased, and that the ankle joint showed rich blood circulation and active bone metabolism.

In the large cysts, the pathology showed the following features consistent with the SPECT/CT findings: (1) a tortuous fibrocystic wall; (2) numerous dense fibroblast proliferations and abundant collagen fibers; (3) granulation tissue and inflammatory cell aggregation around the cyst wall; and (4) chondrogenesis with ossification and active osteogenesis around the necrotic lesion. The pathologically-active chondrogenesis and ossification explain the abnormal concentration of tracers in the cystic cavity on SPECT/CT static fusion images. The presence of osteogenic repair activity within the cystic lesion is secondary to the injury. Focal necrosis of the talar injury occurs in conjunction with a marked congestive and inflammatory response, active fibrous and bone repair, and rapid formation of a sclerotic shell of protective new bone around the cystic lesion. It maintains the overall integrity and normal morphology of the articular surface of the talus under considerable stress. This is in contrast to the deformation of the femoral head caused by massive osteonecrosis in aseptic femoral head necrosis. However, many questions about the definite cause of cystic degeneration after talar injury and the process of its repair still remain for our further research. At present, it is still the best clinical treatment option, being minimally invasive and providing a means for bone grafting to revascularize and provide osteoinduction and osteoconduction [27, 28], thereby enhancing local support and improving ankle stability.

The causes of OLT formation are a popular issue of interest to many scholars, and these causes may be multifaceted. Cystic lesions are associated with trauma and are a part of osteochondral damage. Continuous small cystic lesion formation is typically distributed over the joint surface and occurs where the most force is exerted. When external forces are less than the shear forces that the cartilage can withstand but greater than those that the subchondral bone can withstand, a fracture or microfracture of the subchondral bone will occur in the presence of cartilage. Furthermore, the local microenvironment is altered, resulting in a focal necrotic capsular lesion. OLS differs from ischemic necrosis in that the talus is rich in blood flow and osteogenic activity, allowing for immediate repair and reconstruction. For treatment, minimally invasive surgery that takes full advantage of this and performs drilling combined with bone grafting is the recommended method. In conclusion, SPECT/CT three-phase bone imaging is advantageous for identifying the cause of talar injury, while SPECT/CT delayed imaging is advantageous for observing the location, extent of involvement, classification, and repair of cystic lesions in the talus.

## Data Availability

The datasets generated and/or analyzed during the current study are not freely available because of the protection of patient privacy, but are available from the corresponding author on reasonable request.

## Abbreviations

OLT: Osteochondral lesions of the talus
CT: Computed tomography
